# Copy Number Variations in Alternative Splicing Gene Networks Impact Lifespan

**DOI:** 10.1371/journal.pone.0053846

**Published:** 2013-01-30

**Authors:** Joseph T. Glessner, Albert Vernon Smith, Saarene Panossian, Cecilia E. Kim, Nagahide Takahashi, Kelly A. Thomas, Fengxiang Wang, Kallyn Seidler, Tamara B. Harris, Lenore J. Launer, Brendan Keating, John Connolly, Patrick M. A. Sleiman, Joseph D. Buxbaum, Struan F. A. Grant, Vilmundur Gudnason, Hakon Hakonarson

**Affiliations:** 1 Center for Applied Genomics, The Children’s Hospital of Philadelphia, Philadelphia, Pennsylvania, United States of America; 2 Icelandic Heart Association, Heart Preventive Clinic and Research Institute, Kopavogur, Iceland; 3 University of Iceland, Reykjavik, Iceland; 4 Laboratory of Molecular Neuropsychiatry, Department of Psychiatry, Mount Sinai School of Medicine, New York, New York, United States of America; 5 Laboratory of Epidemiology, Demography, and Biometry, National Institute on Aging, National Institutes of Health, Bethesda, Maryland, United States of America; 6 Division of Genetics, The Children’s Hospital of Philadelphia, Philadelphia, Pennsylvania, United States of America; 7 Department of Pediatrics, University of Pennsylvania Perelman School of Medicine, Philadelphia, Pennsylvania, United States of America; Johns Hopkins University, United States of America

## Abstract

Longevity has a strong genetic component evidenced by family-based studies. Lipoprotein metabolism, FOXO proteins, and insulin/IGF-1 signaling pathways in model systems have shown polygenic variations predisposing to shorter lifespan. To test the hypothesis that rare variants could influence lifespan, we compared the rates of CNVs in healthy children (0–18 years of age) with individuals 67 years or older. CNVs at a significantly higher frequency in the pediatric cohort were considered risk variants impacting lifespan, while those enriched in the geriatric cohort were considered longevity protective variants. We performed a whole-genome CNV analysis on 7,313 children and 2,701 adults of European ancestry genotyped with 302,108 SNP probes. Positive findings were evaluated in an independent cohort of 2,079 pediatric and 4,692 geriatric subjects. We detected 8 deletions and 10 duplications that were enriched in the pediatric group (P = 3.33×10^−8^–1.6×10^−2^ unadjusted), while only one duplication was enriched in the geriatric cohort (P = 6.3×10^−4^). Population stratification correction resulted in 5 deletions and 3 duplications remaining significant (P = 5.16×10^−5^–4.26×10^−2^) in the replication cohort. Three deletions and four duplications were significant combined (combined P = 3.7×10^−4^−3.9×10^−2^). All associated loci were experimentally validated using qPCR. Evaluation of these genes for pathway enrichment demonstrated ∼50% are involved in alternative splicing (P = 0.0077 Benjamini and Hochberg corrected). We conclude that genetic variations disrupting RNA splicing could have long-term biological effects impacting lifespan.

## Introduction

The idea of extended lifespan has fascinated generations of scholarly thought. Specific diseases have been the focus of much biomedical research rather than overarching longevity which in essence successfully avoids a variety of diseases. The average lifespan of the human population has continued to increase at a slow rate due to medical and technological advances that aim at preventing and treating both acute and chronic diseases and attenuating morbidity and mortality of old age [1]. Identification of underlying causes of early fatality provides information that can facilitate preventive measures. As hypothesis free approach is the gold standard to assay genomic variants for disease states, it is equally important to take a hypothesis free approach to assay longevity, one of the most informative measures of health vs. disease states. This approach also addresses the complication in genetics of pleiotropy (one gene:many diseases) where disease phenotype variability results in insufficient power of single disease association studies.

Model systems have demonstrated that lifespan can be dramatically extended by mutations in conserved pathways that regulate growth, energy metabolism, nutrition sensing, and reproduction [2]. A low activity level of organs in many cases extends lifespan perhaps by reduction of somatic damage and increase of somatic maintenance and repair [2]. Strict diet maintaining just above malnutrition has been shown to extend longevity [3]. The leap from model system to human is substantial given the lack of genetic diversity and protective laboratory environment of model systems. It is more probable that significant longevity was achieved by subtle changes in many genes over the course of evolution, not by single mutations with large effects, which often increase lifespan at a cost to reproduction or survival under stress [4].

Genome instability, macromolecular aggregates, decrease in innate immunity, skin/cuticle morphology changes, decreased mitochondrial function, degenerative loss of skeletal muscle mass and strength, and decreased fitness are highly conserved phenotypes of ageing. Lifelong accumulation of various types of damage, along with random errors in DNA maintenance, might underlie intrinsic ageing. Early findings of mutant *C. Elegans* with extended lifespan [5] and linkage studies [6] showed that longevity could be associated with genetic traits. A meta-analysis of 4 cohorts of individuals surviving over 90 years of age found *MINPP1* (involved in cellular proliferation) as well as *LASS3* and *PAPPA2* to be involved [7]. Genes impacting lipoprotein metabolism [8]–[10], *FOXO* proteins [11]–[12], and insulin/*IGF-1* signaling [13]–[15] in humans have also been associated with lifespan.

Copy number variations (CNVs) are rare losses and gains in DNA sequences that have been importantly implicated in the pathogenesis of various neurodevelopmental and psychiatric diseases [16]–[18]. As opposed to SNP genotypes which have revealed common variants conferring modest relative risk to the individual with the variant, CNVs are often rare variants not observed or extremely rare in a normal control population and conferring high relative risk. SNP arrays have vastly improved the detection of CNVs across the human genome over classical methods of karyotype review under a microscope. While the realm of neuropsychiatric and other system disorders have been explained in part by CNVs, it remains to be determined if there are certain gene classes or networks of genes that are pathogenic or disease-causing in general, and if there are other gene networks that may be protective in the same manner. One way of testing this is to compare CNV states and frequencies between pediatric and geriatric subjects and determine if certain CNVs are lost in the older age group (i.e. suggesting pathogenic impact with shortened lifespan), and if other CNVs are enriched and considered protective. Since the detection of CNVs has greatly improved and continues to improve with simultaneous evaluation of genotype and intensity data with continuous coverage of the genome and differentiating models of the diploid from the CNV state, we have undertaken such comparisons in cohorts of pediatric (0–18) and adult cohort above the age of 67.

## Results

**Figure 1:**
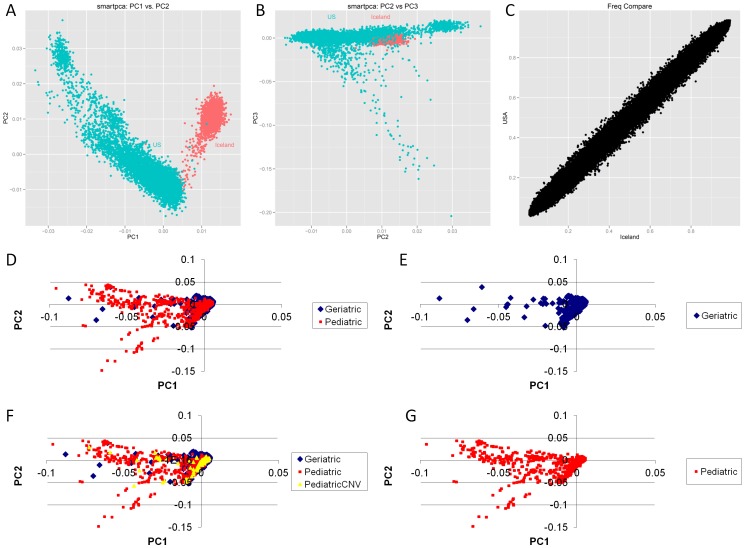
Principle Components Analysis of Pediatric and Geriatric Cohorts. Discovery U.S. Pediatric vs. Icelandic Geriatric A) Principle components (PC) 1 vs. 2 shows distinct clusters likely due to sporadic differential profiles of a specific subset of SNPs between arrays. Since CNV calling is based on multiple neighboring SNPs and differential clustering SNPs are randomly distributed, CNV discovery should not experience significant bias. B) PC2 vs. 3 representing population structure showing some overlap of pediatric and geriatric cohorts C) SNP genotype allele frequency differences genome wide showing close correlation. Replication U.S. Pediatric vs. U.S. Geriatric D) Replication of U.S. pediatric and U.S. geriatric PC1 vs. PC2 showing high overlap unlike panel A U.S. pediatric and Icelandic geriatric E) Geriatric replication cohort in isolation for clarity F) Population structure of pediatric subjects with significantly associated risk CNVs for short lifespan showing broad normal distribution minimizing test statistic inflation for rare variants opposed to tight clustering(37) G) Pediatric replication cohort in isolation for clarity.

The pediatric discovery group included 7,313 children recruited at the Children’s Hospital of Philadelphia (Table 1). The geriatric discovery cohort included 2,701 individuals recruited by the Icelandic Heart Association in the AGES Reykjavik study of 67 years or older. Only samples meeting strictly established data quality thresholds for copy number variation were included in the analysis. Pediatric subjects were genotyped on the Illumina Human Hap550 while geriatric subjects were genotyped on the Illumina HumanCNV370-Duov1.0. To ensure comparability of results, only the intersection set of 302,108 SNPs common to both platforms was evaluated. All arrays used the Illumina Infinium II beadchip technology with standardized reagents, oligos, and experimental protocol to minimize variation between genotyping at different sites. Multiple neighboring SNPs (minimum 3) are required to make a CNV call so one biased SNP in a region will not bias the CNV calling. CNVs were scored with both PennCNV [19] and QuantiSNP [20] for copy number deviating from normal diploid state 2: states 0 and 1 for deletions and 3 and 4 for duplications. We compared frequency of deletions and duplications between pediatric and geriatric subjects to assess significant enrichment of rare recurrent CNVs in either group. Evaluating the SNP genotype data revealed tight clustering of populations at the origin by principle components analysis (PCA) indicative of European ancestry. Unfortunately, low overlap of populations was observed when the pediatric and geriatric cohorts were plotted together (Figure 1A and 1B). Many CNV and genotype associations made in cohorts of European ancestry have shown robust replication in Icelandic cohorts [21]–[26], indicating that CNVs observed in the more broadly-defined European and American Caucasian gene pool are also important in the Icelandic population. The Icelandic cohort is unique in having risk factor assessments earlier in life and detailed late-life phenotypes of quantitative traits [27]. Our rationale for comparing these cohorts was the availability of large pediatric and geriatric populations with extensive phenotype characterization both genotyped on the Illumina microarray. While the PCA analysis clearly shows this comparison to be impacted by population stratification and that PCA cannot be applied as covariates due to this lack of overlap, we believe this comparison can be hypothesis generating in showing if such associated variants can be replicated in an independent population with a very good PCA overlap, but less phenotype depth.

**Table 1:** Discovery and Replication Case:Control Sample Sets.

Cohort	Samples Count	Country of Origin
Discovery CHOP Pediatric	7,313	United States
Discovery IHA Geriatric	2,701	Iceland
Replication CHOP Pediatric	2,079	United States
Replication Geriatric	4,692	United States

Contributing project totals in discovery and replication phases. The totals represent the number of high quality datasets derived from samples.

**Figure 2:**
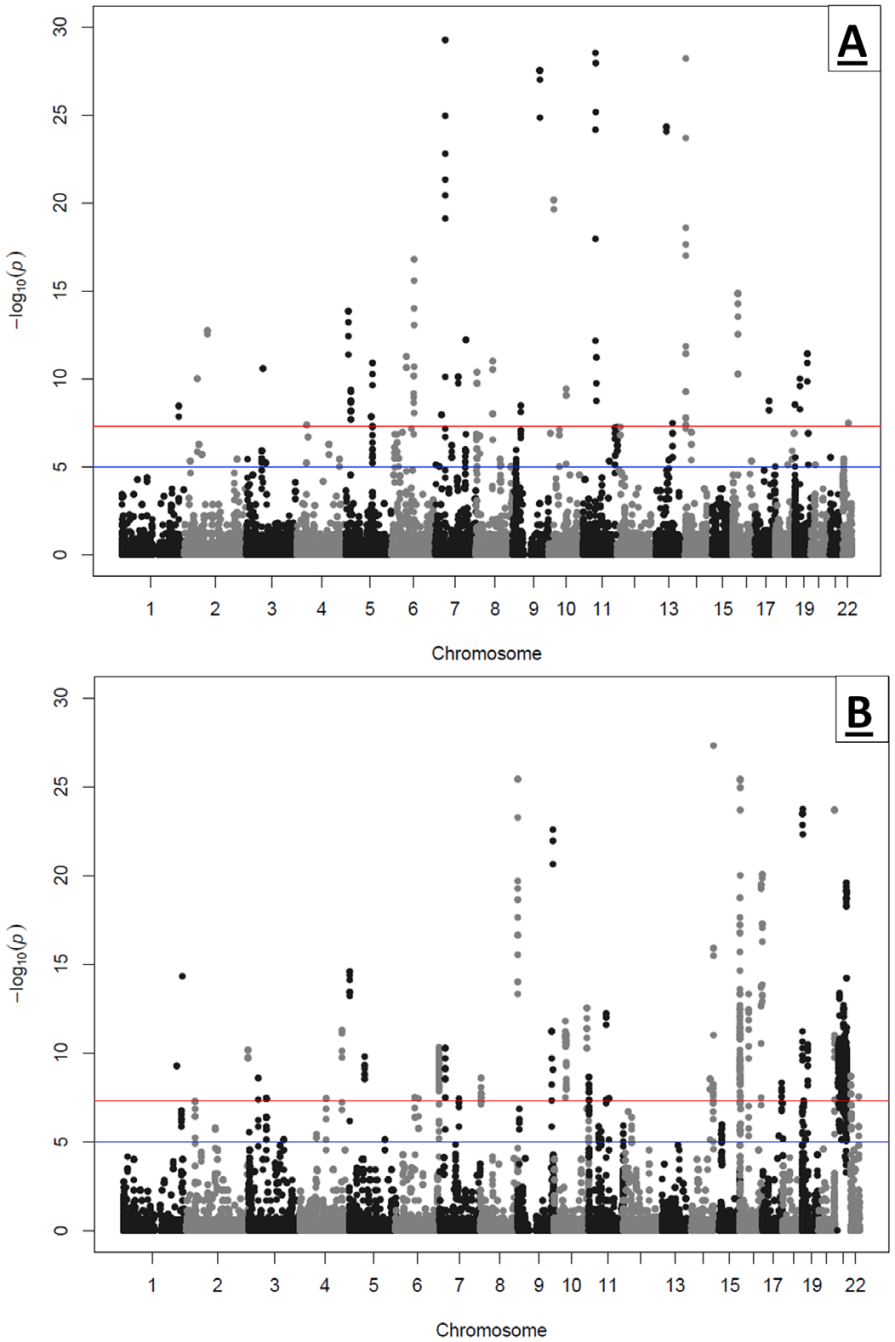
Manhattan Plot of SNP based CNV Statistics. (A)Deletions and (B)Duplications.

To associate CNV loci potentially contributing to shortened lifespan, we applied a segment-based scoring approach that scans the genome for consecutive probes with more frequent copy number changes in pediatric compared to geriatric subjects. The genomic span for these consecutive probes forms common copy number variation regions (CNVRs). We uncovered 101 loci with deletion and 76 with duplication enrichment in the pediatric cohort. Conversely, we identified 90 loci with deletion and 74 with duplication enrichment in the geriatric cohort (Figure 2). After raw data QC and genomic context review, a high confidence discovery set of 55 deletions and 40 duplications that were significantly enriched in the pediatric cohort resulted while 53 deletions and 43 duplications were enriched in the geriatric cohort. These filtering criteria included exclusion of telomere, centromere, CNV boundary uncertainty, extreme GC content, poor SNP coverage, and CNVR sample bias. CNVR sample bias refers to the same sample contributing to the association signal of many different significant CNVRs, despite up-front sample quality control, often due to atypical intensity wave patterns.

We next sought to independently replicate these CNV findings in additional pediatric and geriatric subjects. CNVs were called for 2,079 young age subjects from independent pediatric cohorts all of which were recruited in the U.S.A and genotyped on the Illumina Infinium Human Hap550. We compared the CNV frequency in young with an independent cohort of 4,692 older subjects (over 50), all of which were recruited in the U.S.A. and genotyped on the Illumina Infinium Human660W-Quad. We replicated in the same direction 11 deletions and 10 duplications that were significantly enriched in the pediatric cohort, while 1 duplication was enriched in the geriatric cohort. As shown in Figure 1, in contrast to the Icelandic geriatric vs. U.S. pediatric PCA plot (panel 1A), the replication U.S. geriatric vs. U.S. pediatric did show strong overlap (panel 1D) indicating comparable population structure. Furthermore, we were able to correct for any residual population structure using the first three components of the PCA as covariates for logistic CNV association. This gives the unique opportunity to test replication of associated loci between non-overlapping PCA populations which cannot be corrected by covariates with well overlapping PCA populations controlled by covariates. We can also assess replication between Illumina array versions for consistent CNV detection. We believe leveraging existing data with a variety of variations may lead to associations more likely to remain significant by further studies where these variations are often manifest in addition to data processing variations which we were able to control by applying consistent processing across all data.

**Figure 3:**
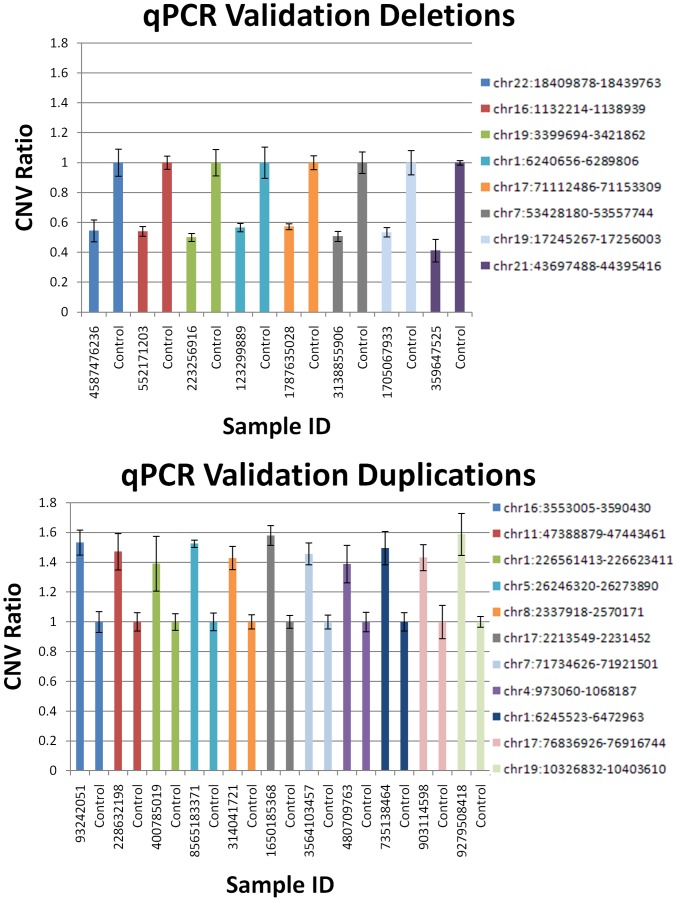
Independent Technology Validation of Presence of CNV Events to Confirm CNVs Detected by Illumina Array. Error bars denote the standard deviation of quadruplicate runs.

To assess the reliability of our CNV detection method, we experimentally validated all the significant CNVRs using an independent wet lab method, quantitative real time polymerase chain reaction (qPCR) (Figure 3) on a randomly selected samples with a CNV at each associated locus and samples without a CNV to normalize the measurement. This yielded a final confident set of 8 deletions and 10 duplications that were significantly enriched in the pediatric cohort (Table 2) while 1 duplication was enriched in the geriatric cohort (Table 3).

**Table 2:** CNVs Enriched in Pediatric Individuals.

CNVR hg18	CHOP Pediatric	IHA Geriatric	P Discovery	Replication Pediatric	Replication Geriatric	P PCA Corrected Replication	P Combined	Gene	Type
chr8∶2337918–2570171	87	4	3.33E-08	30	24	0.001406	**0.00037**	*AK128880,BC045738*	Dup
chr22∶18409878–18439763	42	0	3.89E-06	9	4	0.00487	**0.003862**	*C22orf25,DKFZp761P1121*	Del
chr16∶3553005–3590430	60	1	1.37E-07	16	0	0.9961	**0.008209**	*BTBD12,NLRC3*	Dup
chr1∶226561413–226623411	50	0	1.87E-07	7	0	0.9975	**0.00924**	*KIAA1639,OBSCN*	Dup
chr19∶17245267–17245267	19	1	0.02286	12	3	5.16E-05	**0.018451**	*HSPC142/BABAM1*	Del
chr1∶6240656–6289806	26	0	0.0005	8	3	0.002979	**0.020119**	*ACOT7,BACH,GPR153*	Del
chr11∶47388879–47443461	66	4	9.00E-06	16	0	0.9965	**0.038865**	*PSMC3,RAPSN,SLC39A13*	Dup
chr7∶53428180–53557744	29	0	0.00019	8	5	0.1969	0.064854	*FLJ45974* *	Del
chr17∶71112486–71153309	20	1	0.02352	9	1	0.002534	0.076432	*LOC643008,MYO15B,RECQL5*	Del
chr21∶43697488–44395416	14	0	0.01601	5	1	0.007178	0.096104	*AGPAT3,C21orf125,C21orf33,C21orf84,CSTB,HSF2BP,LOC284837,PDXK,PWP2,RRP1,RRP1B,TRAPPC10*	Del
chr4∶973060–1068187	25	1	0.00626	9	1	0.02017	0.099286	*FGFRL1,IDUA,LOC285498,RNF212,SLC26A1*	Dup
chr7∶71734626–71921501	37	3	0.00369	8	1	0.0426	0.10708	*MGC87315*	Dup
chr17∶2213549–2231452	25	0	0.0005	7	0	0.9981	0.15837	*KIAA0397,RUTBC1*	Dup
chr16∶1132214–1138939	38	3	0.00246	8	0	0.9979	0.26546	*CACNA1H* *	Del
chr19∶10326832–10403610	14	0	0.01601	4	0	0.9986	0.46396	*CDC37,PDE4A,TYK2*	Dup
chr19∶3399694–3421862	22	2	0.03849	10	0	0.9974	0.5864	*NFIC*	Del
chr1∶6245523–6472963	11	0	0.04318	12	0	0.997	0.60362	*ACOT7,ESPN,HES2,PLEKHG5,TNFRSF25*	Dup
chr17∶76836926–76916744	11	0	0.04318	9	0	0.9977	0.60373	*C17orf55,MGC15523,TMEM105*	Dup

* Gene not overlapped so closest proximal gene annotated. Gene delimiters were defined based on UCSC genes table reference including exons and introns. Any direct overlap of any segment of the gene delimiters is considered a hit such that complete overlap of the gene is not required. Combined p-values were calculated using Fisher’s method.

**Table 3:** CNVs Enriched in Geriatric Individuals.

CNVR hg18	CHOPPediatric	IHAGeriatric	P Discovery	ReplicationPediatric	ReplicationGeriatric	P PCA CorrectedReplication	P Combined	Gene	Type
chr5∶26,246,320–26,273,890	1	7	0.00063	0	24	0.9963	0.17091	CDH9*	Dup

To fully correct for population stratification, in addition to multi-dimensional scaling, we performed principal component analysis (PCA) on the genotypes and used the resulting first three components as covariates of logistic test CNV association in the replication cohort. CNV events in our study are rare and arise randomly shown by evaluating the spatial distribution of samples having a risk CNV on the PCA plot revealing a Gaussian (at minimum uniform due to few data points) distribution which indicating minimal test statistic inflation (even less than common variants) as opposed to a small, sharply defined region [37] (Figure 1F). We verified that population stratification was fully controlled for based on a genomic inflation factor of 1.0. Eight of eighteen pediatric enriched CNV loci remained significant (p<0.05) following PCA population stratification correction (five deletions and three duplications; see Table 2). These results indicate that, while population stratification did indeed influence nominal p-value of the associated rare CNV variants in the discovery cohort, it could be corrected in the independent replication cohort, leaving a number of associated loci that replicated.

Given the diverse etiology of diseases and more generally, lack of fitness in an evolutionary context, the genes underlying the broad consideration of ageing are similarly diverse. Single significant loci are certainly of interest to the common genomic CNVs resulting in specific genes to study. However, strong confidence in the result set generated can be achieved by observing the same biological system being perturbed by multiple independently significant loci. Motivated by this, genes directly overlapped by associated CNVs were prepared as a single list and non-RefSeq hypothetical gene IDs were removed. This list was entered into DAVID functional annotation enrichment tool in contrast with a background representing genome-wide regions covered by the array. Taking into account the size of different genes and the gene family size of different annotations, the enrichment of our CNV impacted list was assigned a p-value with Benjamini and Hochberg correction for multiple testing. Functional annotations from multiple databases were used including KEGG and GO (gene ontology). Functional categories were reviewed for genes contributing from distinct genomic regions to reject enrichment of closely clustered gene families.

To identify potential functional biases specific to CNVs observed at significantly higher frequency in young individuals, we evaluated clustering into specific functional categories using DAVID [28], [29] (Database for Annotation, Visualization, and Integrated Discovery). We found significant overrepresentation of alternative splicing genes impacted by the CNVs. To limit contribution of regions with gene families of related function, each CNV loci was limited to contributing one gene to a functional cluster, done by referencing resulting gene clusters back to the input genes from each CNV region. Among the alternative splicing genes are *AGPAT3, BTBD12, NLRC3, RECQL5, SCAPER, ACOT7*, *C19orf62*, *C21orf33*, *C22orf25*, *ESPN*, *HES2*, *LUZP2*, *NFIC*, *OBSCN*, *PDE4A*, *PLEKHG5*, *PLXDC1*, *KCNT1*, *PDXK*, *RAPSN*, *RRP1B*, *RNF212*, *SGSM2*, *SLC38A10*, *SLC39A13*, and *TNFRSF25* all of which were significantly enriched in the young age group (*P* = 0.0077 Benjamini and Hochberg corrected), suggesting that genetic variations that disrupt RNA splicing may have long-term biological effects on human lifespan.

## Discussion

Limited nutrition, somatic maintenance and growth are pathways to longevity. Emphasis on somatic maintenance is more important than early growth and reproduction. Post-transcriptional modification of mRNA is an important mechanism which results in a variety of protein isoforms and occurs in at least 80% of human genes, and known to harbor variations that have been associated with human disease [30]. It is therefore of interest that 50% of the genes impacted by CNV loci significantly enriched in young and replicated in an independent cohort were responsible for alternative splicing, suggesting that genetic variants in these gene networks may be pathogenic and disease causing in a more global way than previously thought.

Alternative splicing is an abundant violation of the one gene one protein theory initially regarded. The exons of an mRNA can be edited producing a variety of combinations which result in a variety of protein isoforms. This mechanism allows for a great diversity of protein products based on the same DNA code and branches out gene families much the way ancestral duplications extends gene families in DNA. Proteins responsible for alternative splicing bind to specific RNA sequences to promote or repress splicing.

SNPs in the RNA editing genes *ADARB1* and *ADARB2* were associated with extreme old age in a United States based study of centenarians with replication to four other ethnic backgrounds [31]. DNA maintenance is of fundamental importance throughout the lifespan and is under assault by environmental conditions such as sunlight and chemical exposures. *BTBD12* and *BABAM1* are part of a multi-protein complex containing enzymes involved in DNA maintenance and repair of serious damage such as collapsed replication forks and double-strand breaks (DSBs) [32]. Of note, *BABAM1* is the most highly significant CNV associated locus following full statistical correction of population stratification (p = 5.16×10^−5^).

**Figure 4:**
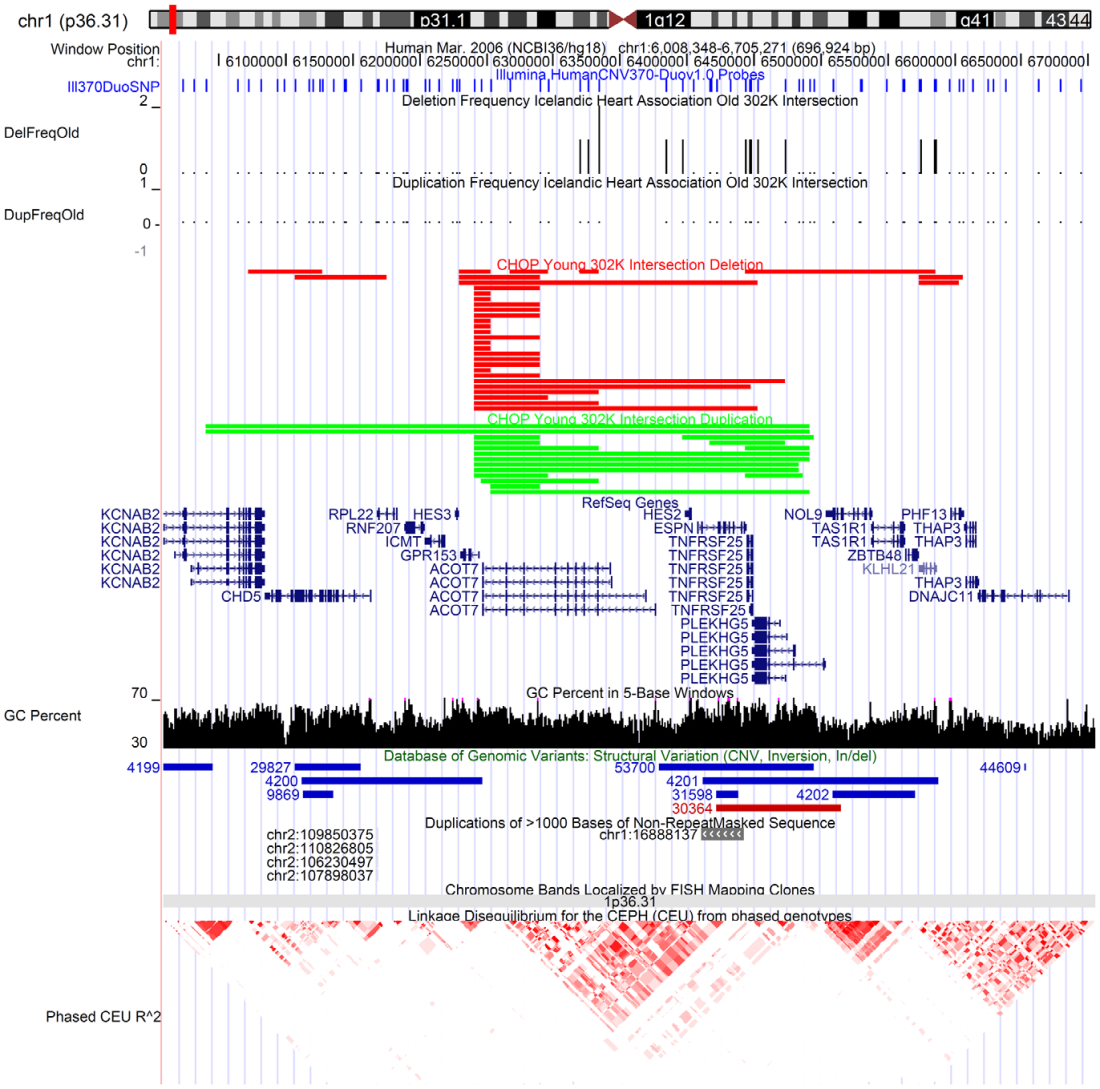
Regions of CNV in Young Individuals observed at low levels in Older Individuals. *ACOT7* locus shows significant excess of deletions and duplications in young individuals. Blue lines indicate SNP marker coverage to resolve CNV boundaries. Histogram shows the number of subjects with deletion and duplication CNVs in the Icelandic older population (very low). The red and green boundaries show individual CNVs observed in specific young samples from CHOP. Genomic region references including GC percent, RefSeq Genes, and Database of Genomic Variants are provided for reference.

*ACOT7* is involved with biosynthesis of unsaturated fatty acids and decreased expression is associated with mesial temporal lobe epilepsy. Young individuals showed significantly higher frequency of both deletions and duplications of this locus compared to older individuals (Figure 4). Nuclear factor kappa B (NFKB1) signaling pathway is a fundamentally important protein complex that controls the transcription of DNA and responds to external factors such as stress, cytokines, free radicals, ultraviolet radiation, oxidized LDL, and bacterial or viral antigens. *PLEKHG5* activates the NFKB1 signaling pathway. *TNFRSF25* encodes a receptor that has been shown to stimulate NF-kappa B activity and regulate cell apoptosis. The TNF-receptor signaling pathway is critically involved in the pathogenesis of inflammatory bowel disease and rheumatoid arthritis [33]. Such a pivotal gene is an example of autoimmune disease and strong immunity aiding survival in early age but early death as a consequence. Increased recombination rate has been shown to occur in older age mothers. *RNF212* is essential for recombination & chiasma formation in C elegans [34]. A CNV in a gene controlling recombination could lead to genome instability and excessive recombination with more chances for errors.

**Figure 5:**
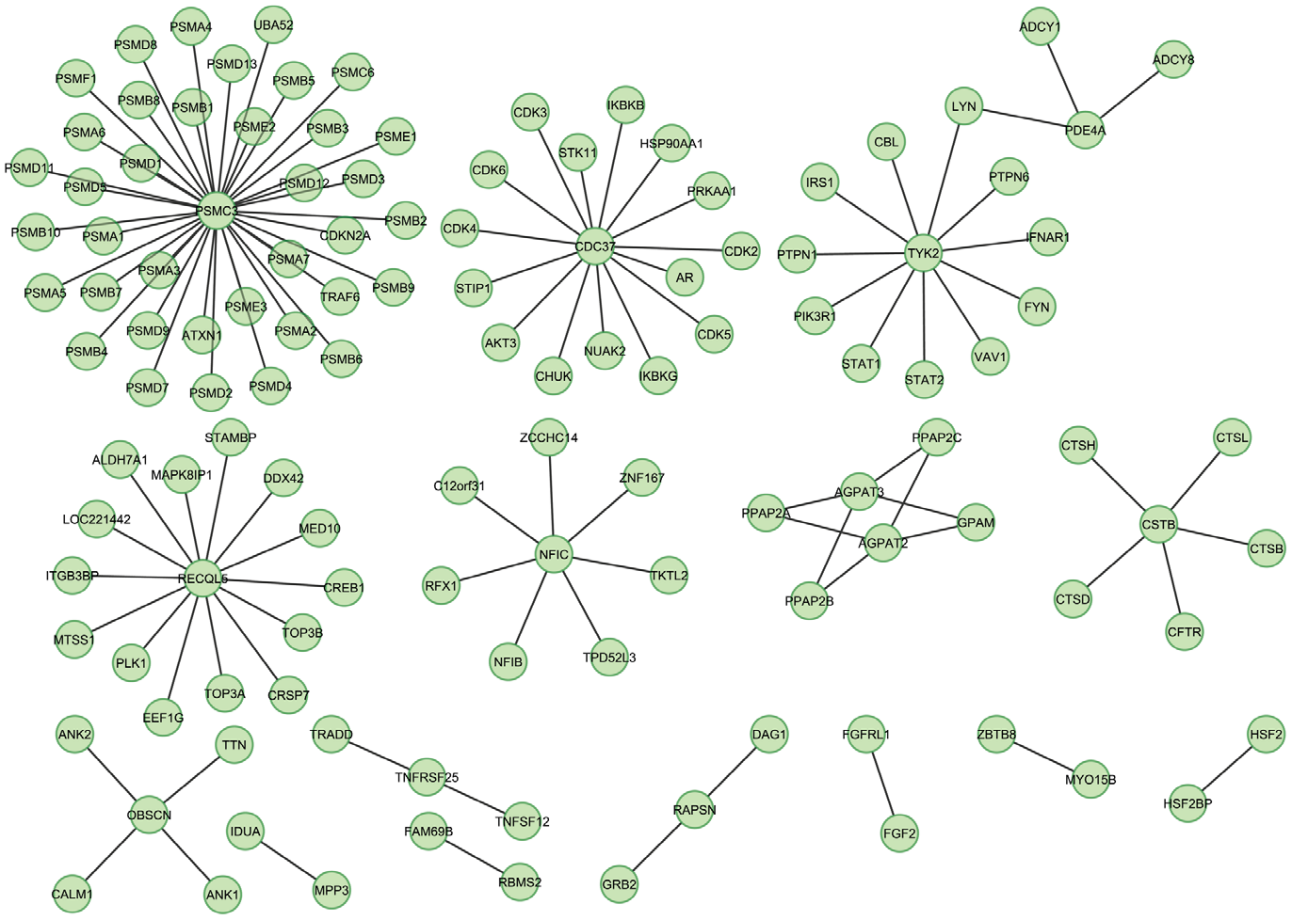
Representative Interactions of the Lifespan Longevity Associated Genes Identified. Gene-gene interactions of independently significant loci. Additional genes implicated by interacting with genes in significantly associated longevity loci. Alternative splicing gene function annotation enrichment of significant loci suggests diverse genetic perturbation with a common biological role. Extension of this functional category to other genes annotated by functional studies with interactions to associated genes implicates potential for screening diverse etiology.

Given that typical cause of death among different individuals is highly heterogeneous from a clinical perspective, the underlying genetic causes of premature death or attenuated longevity are likely to have similarly variegated set of genes. Therefore, based on the specific loci found significantly associated with lifespan, more integrative systems biology is possible leveraging protein-protein interactions using Cytoscape [35] (Figure 5). Profiling expressed sequence tags (ESTs), smaller numbers of cDNA sequences assayed by microarrays and RNA-Seq has allowed for more complete profiling of alternative splicing [36]. Continuing study on different tissues of the body coupled to CNV findings through high-throughput sequencing approaches in the future can help elucidate underlying mechanisms of ageing.

This study represents the first genome-wide population based copy number variation study of human longevity, applying a unique study design to identify the pathogenic nature of CNVs at a global scale in human. The use of the relatively large cohorts assembled here was essential, both to discover and to confirm the findings and demonstrates the potential of genome-wide association in complicated polygenic ageing. This type of unbiased study has discovered many novel targets that may underlie short lifespan. We have focused on robustly identifying CNVs observed in a large sample of pediatric and comparing those observations to a large geriatric sample to see which CNVs limit the lifespan from reaching old age. This is distinct from the question of longevity to extremely late age but CNV occurrence in these genes reduce longevity and which effects need to be counteracted to produce exceptional longevity. These genetic variations can be screened in a clinical setting to prognosticate future premature death and prophylactic measures taken with a potential impact on healthcare management in the future.

## Materials and Methods

### Ethics Statement

This research was approved by the Institutional Review Board of the Children’s Hospital of Philadelphia. All subjects were recruited and signed written informed consent if age 18 or older. Parents signed written consent on the behalf of minors/children age 0–17 and the child signed a written assent if 7–17 years of age. The Data Protection Commission of Iceland and the National Bioethics Committee of Iceland approved this research on adult samples. The appropriate written informed consent was obtained for all adult sample donors.

### Study Subjects

A cohort of healthy children under the age of 19 recruited within the Health Care Network of the Children’s Hospital of Philadelphia was compared with adult subjects above the age of 67 (average age 76), recruited for the AGES-Reykjavik study [27]. The replication cohort was composed of young previously published in the context of autism [17] and older individuals accessed from dbGaP, including the Personalized Medicine Research Project (PMRP). The average age of the children was 8.6 years and average age of the adults was 60 years, with equal numbers of males and females.

### Illumina Infinium Assay for CNV Discovery

We performed high-throughput, genome-wide SNP genotyping, using the InfiniumII HumanHap550 BeadChip technology (Illumina San Diego CA), at the Center for Applied Genomics at CHOP. The genotype data content together with the intensity data provided by the genotyping array provides high confidence for CNV calls. Importantly, the simultaneous analysis of intensity data and genotype data in the same experimental setting establishes a highly accurate definition for normal diploid states and any deviation thereof. To call CNVs, we used the PennCNV algorithm, which combines multiple sources of information, including Log R Ratio (LRR) and B Allele Frequency (BAF) at each SNP marker, along with SNP spacing, a trained hidden Markov model, and population frequency of the B allele to generate CNV calls. The intersection set of 302,108 probes common to the Illumina 550K: 532,898 probes and Illumina 370 Duo: 370,405 probes was used to make datasets as comparable as possible.

### CNV Quality Control

We calculated Quality Control (QC) measures on our HumanHap660 GWAS data based on statistical distributions to exclude poor quality DNA samples and false positive CNVs. The first threshold is the percentage of attempted SNPs which were successfully genotyped. Only samples with call rate >98% were included. The genome wide intensity signal must have as little noise as possible. Only samples with the standard deviation (SD) of normalized intensity (LRR) <0.30 were included. All samples must have clear European ethnicity based on Eigenstrat smartPCA scoring and all other samples were excluded. Wave artifacts roughly correlating with GC content resulting from hybridization bias of low full length DNA quantity are known to interfere with accurate inference of copy number variations. Only samples where the GC wave factor of LRR |GCWF|<0.05 were accepted. If the count of CNV calls made by PennCNV exceeds 100, the DNA quality is usually poor. Thus, only samples with CNV call count <100 were included. Any duplicate samples (such as monozygotic twins) had one sample excluded.

### Statistical Analysis of CNVs

CNV frequency between cases and controls was evaluated at each SNP using Fisher’s exact test. We only considered loci that were significant between cases and controls (p<0.05) where cases in the discovery cohort had the same variation, replicated in an independent cohort or were not observed in any of the control subjects, and validated with an independent method. We report statistical (p-value) local minimums to narrow the association in reference to a region of nominal significance including SNPs residing within 1 Mb of each other. Resulting significant CNVRs were excluded if they met any of the following criteria: i) residing on telomere or centromere proximal cytobands; ii) arising in a “peninsula” of common CNV arising from variation in boundary truncation of CNV calling; iii) genomic regions with extremes in GC content which produces hybridization bias; or iv) samples contributing to multiple CNVRs. A peninsula is defined as a false positive association arising from a region of common CNV extending variably due to variability in probe performance and variability in samples. In other words, the specific significant subregion is confounded by contributing calls also extending to a non-significant subregion.

To fully correct for population stratification, we performed (PCA) on the genotypes and used the resulting first three components as covariates of the logistic test for CNV association using Plink.

Combined p-values were calculated using Fisher’s method
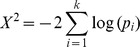
where *p_i_* is the p-value for the *i*th study. Under the null hypothesis, *X* ^2^ follows a chi-squared distribution with 2*k* degrees of freedom, where *k* is the number of studies. In this case, there were two studies yielding a chi-squared distribution with four degrees of freedom.

To inform multiple testing correction, CNV filtering steps have been performed as part of the analysis. Firstly, it is important to note that of the intersection set of 302,108 SNPs on the Illumina array, 3,911 (1.295%) showed deletion and 8,830 (2.923%) showed duplication in at least eleven or more unrelated cases in the discovery cohort (frequency ≥0.150%). 41,392 (13.701%) deletion and 45,050 (14.912%) duplication SNPs were observed in at least two individuals. The threshold of three cases harboring a given CNV is selected because it is the minimal case frequency to provide minimal expectation of frequency differences between cases and controls to yield nominal statistical significance and reproducibility for the calls in a given region. We find this upfront exclusion to be very similar to the inclusion threshold of 1% minor allele frequency in GWA SNP genotype studies. These SNPs were collapsed into 101 deletion and 76 duplication CNVRs based on necessary multiple neighboring SNP signals to call a CNV and resulting redundancy of individual SNP statistics. This results in a total of 171 tests being performed corresponding to a multiple testing correction bar of p = 2.92E-4 close to the p = 5E-4 bar we have seen previously.

### Gene Category Enrichment

Given the diverse etiology of diseases and more generally, lack of fitness in an evolutionary context, the genes underlying the broad consideration of ageing are similarly diverse. Single significant loci are certainly of interest to the common genomic CNVs resulting in specific genes to study. However, strong confidence in the result set generated can be achieved by observing the same biological system being perturbed by multiple independently significant loci. Motivated by this, genes directly overlapped by associated CNVs were prepared as a single list and non-RefSeq hypothetical gene IDs were removed. This list was entered into DAVID functional annotation enrichment tool in contrast with a background representing genome-wide regions covered by the array. Taking into account the size of different genes and the gene family size of different annotations, the enrichment of our CNV impacted list was assigned a p-value with Benjamini and Hochberg correction for multiple testing. Functional annotations from multiple databases were used including KEGG and GO (gene ontology). Functional categories were reviewed for genes contributing from distinct genomic regions to reject enrichment of closely clustered gene families.
